# Miconazole and phenothiazine hinder the quorum sensing regulated virulence in *Pseudomonas aeruginosa*

**DOI:** 10.1038/s41429-024-00731-5

**Published:** 2024-05-09

**Authors:** Amany I. Gad, Amira M. El-Ganiny, Ahmed G. Eissa, Nada A. Noureldin, Shaimaa I. Nazeih

**Affiliations:** 1 Microbiology and Immunology Department, Faculty of Pharmacy and Drug Technology, Egyptian Chinese University, Cairo, 11786 Egypt; 2 Microbiology and Immunology Department, Faculty of Pharmacy, Zagazig University, Zagazig, 44519 Egypt; 3 Medicinal Chemistry Department, Faculty of Pharmacy, Zagazig University, Zagazig, 44519 Egypt

**Keywords:** Antimicrobials, Pathogenesis

## Abstract

Antibiotic resistance is a major health problem worldwide. *Pseudomonas aeruginosa* is a Gram-negative pathogen with an arsenal of virulence factors and elevated antimicrobial resistance. It is a leading cause of nosocomial infections with high morbidity and mortality. The significant time and effort required to develop new antibiotics can be circumvented using alternative therapeutic strategies, including anti-virulence targets. This study aimed to investigate the anti-virulence activity of the FDA-approved drugs miconazole and phenothiazine against *P. aeruginosa*. The phenotypic effect of sub-inhibitory concentrations of miconazole and phenothiazine on biofilm, pyocyanin, protease, rhamnolipid and hemolysin activities in PAO1 strain was examined. qRT-PCR was used to assess the effect of drugs on quorum-sensing genes that regulate virulence. Further, the anti-virulence potential of miconazole and phenothiazine was evaluated in silico and in vivo. Miconazole showed significant inhibition of *Pseudomonas* virulence by reducing biofilm-formation approximately 45–48%, hemolytic-activity by 59%, pyocyanin-production by 47–49%, rhamnolipid-activity by approximately 42–47% and protease activity by 36–40%. While, phenothiazine showed lower anti-virulence activity, it inhibited biofilm (31–35%), pyocyanin (37–39%), protease (32–40%), rhamnolipid (35–40%) and hemolytic activity (47–56%). Similarly, there was significantly reduced expression of *RhlR, PqsR, LasI* and *LasR* following treatment with miconazole, but less so with phenothiazine. *In-silico* analysis revealed that miconazole had higher binding affinity than phenothiazine to LasR, RhlR, and PqsR QS-proteins. Furthermore, there was 100% survival in mice injected with PAO1 treated with miconazole. In conclusion, miconazole and phenothiazine are promising anti-virulence agents for *P. aeruginosa*.

## Introduction

*Pseudomonas aeruginosa* is a Gram-negative pathogen and one of the major causes of nosocomial infections especially in immunocompromised patients. *P. aeruginosa* infections include bacteremia, dermatitis, urinary tract infections and respiratory tract infections, particularrlyin cystic fibrosis patients [1]. The severity of *P. aeruginosa* infections is associated with intrinsic and acquired antibiotic resistance, in addition to its arsenal of virulence factors. *P. aeruginosa* virulence factors include the production of toxins, extracellular invasive enzymes, and secondary metabolites such as pyocyanin and rhamnolipids, as well as resistant biofilm formation [2].

The production of virulence factors and biofilm formation in *P. aeruginosa* are under control of the quorum sensing (QS) machinery. *P. aeruginosa* have multiple QS-systems, of which the most-studied are the LasI/R and RhlI/R QS, which rely on binding of acyl homoserine lactone auto-inducers (AIs). A third QS-system employs alkyl quinolone molecules known as Pseudomonas Quinolone Signal (PQS) as an auto-inducer [3]. These three QS-circuits share overlapping regulators, with the Las signal commanding the QS-circuit which activates both Rhl and Pqs [4]. Furthermore, the Las system governs the production of the extracellular protease LasB, a zinc metalloprotease having proteolytic activity against different tissue substrates, considered a virulence indicator in *P. aeruginosa* [5].

Both Rhl and Pqs systems control the production of pyocyanin [4], a blue-green pigment with redox activity that causes damage to DNA and other host cell components leading to cell lysis [6]. Pyocyanin production is considered a core hallmark of *Pseudomonas* infections [3]. Rhamnolipids are surfactants, also related to *P. aeruginosa* lung pathogenesis based on degradation and disruption of tight junctions of the respiratory epithelium [6]. The hemolysin enzymes of *P. aeruginosa* are responsible for the inflammatory response, damage to the host cells and the inhibition of neutrophil defences. In addition, *P. aeruginosa* releases a wide range of extracellular proteases that cleave the peptide bonds of host proteins [7].

The World Health Organization (WHO) has designated *P. aeruginosa* as a “critical-priority” bacteria for the development of new therapies. New solutions for *P. aeruginosa* infections include vaccines, iron chelator, bacteriophages, and anti-virulence agents [8, 9]. Anti-virulence factors can disarm bacterial pathogens without affecting viability therefore reducing development of bacterial resistance [10]. Furthermore, drug repurposing (finding new therapeutic uses for approved drugs) has emerged as an alternative to the synthesis of de novo antibiotics, since the development of new antibiotics is lengthy and costly [11]. Recently, several U.S. food and drug administration (FDA)-approved drugs have been investigated by our lab for their antimicrobial and anti-virulence activities [12–17].

The FDA-approved anti-psychotic drug phenothiazine and its derivatives (e.g. chlorpromazine) showed antibacterial activity and efflux inhibitory properties against Gram positive and Gram negative pathogens, including *P. aeruginosa*, and hence can be used as antibiotic adjuvants [18]. In addition, chlorpromazine had a QS-inhibitory activity in *Chromobacterium violaceum* and *Serratia marcescens* reporter strains [19], and the antifungal drug miconazole has anti-virulence activity by reducing the expression of virulence genes in *P. aeruginosa* [20].

In this study, phenothiazine and miconazole were screened for their anti-virulence potential against *P. aeruginosa* QS-regulated virulence factors, which include pyocyanin, hemolysin, rhamnolipids, protease production and biofilm formation. The approach used both phenotypic and genotypic techniques, along with in silico and in vivo approaches.

## Materials and methods

### Determination of the minimum inhibitory concentrations (MICs) of miconazole and phenothiazine

The MICs of miconazole and phenothiazine were determined by the agar dilution method according to clinical laboratory standard institute (CLSI) guidelines [21]. Different dilutions of miconazole and phenothiazine solutions were prepared and mixed with molten Mueller Hinton agar (MHA) at 50 °C and poured into Petri dishes. Colonies from an overnight culture on MHA were transferred into Mueller Hinton broth (MHB) and incubated at 37 °C for 24 h, and a bacterial suspension of at 0.5 McFarland standard ( ~ 1 × 10^8^ colony forming unit (CFU) ml^−1^) was prepared. The suspension was further diluted 1:10 in sterile distilled water and 1 μL of this suspension (contained 10^4^ CFU ml^−1^) spotted into the surface of MHA plates containing drug dilutions. Drug-free plates inoculated with bacteria served as a positive control, and the plates incubated for 16–20 h at 37 °C. The MIC was recorded as the lowest concentration of the drugs that completely inhibited visible bacterial growth.

### The effect of sub-inhibitory concentration of miconazole and phenothiazine on the viability of *P. aeruginosa*

The effect of miconazole and phenothiazine on *P. aeruginos*a growth and viability was tested [22]. *P. aeruginos*a PAO1 was grown in fresh MHB in the presence and absence of 1/4 and 1/8 MIC of miconazole and phenothiazine. Following incubation at 37 °C overnight, the optical densities (ODs) of bacterial cultures (treated or untreated) were measured at as OD at 600 nm (OD_600_) using Biotek spectrophotometer (Winooski, VT, USA).

### Quantitative assessment of biofilm inhibition by tested drugs at sub-MIC

The biofilm formation was assessed as described previously [23], with some modifications. Briefly, bacterial suspensions were prepared from overnight cultures of PAO1 in tryptone soy broth (TSB) and adjusted to a turbidity of 0.5 McFarland standard. Aliquots of 200 μl bacterial suspension were transferred to the wells of a 96-well microtiter plate in the presence and absence of tested drugs (miconazole and phenothiazine) and incubated for 48 h at 37 °C. The broth was then decanted gently, the plate washed with distilled water, and then left to dry in air. The biofilm was fixed for 20 min with 200 µl of 99% methanol, then stained for 15 min using 200 μl crystal violet solution (1%). The plate was washed prior to solubilization with 200 μl of 33% glacial acetic acid. Finally, the absorbance of the solubilized dye was measured (Biotek spectrophotometer) at 570 nm (A_570_) and used to assess the strength of the biofilm (drug-treated or untreated). Each treatment was in triplicate

### Pyocyanin inhibition assay

Pyocyanin pigment production was assayed according to the method of Das and Manefield [24]. Briefly, an overnight culture of PAO1 in Luria*-*Bertani (LB) broth was prepared and diluted to a turbidity of 0.3–0.4 at OD_600_ and 50 μl of the diluted suspension was used to inoculate 5 ml of LB broth, with and without either miconazole or phenothiazine (at 1/4 and 1/8 MIC). The cultures were incubated at 37 °C for 48 h, the tubes were centrifuged at 10,000 rpm for 10 min at 4 °C. The pyocyanin pigment in the supernatant was quantified by measuring A_691_ (Biotek spectrophotometer).

### Rhamnolipid inhibition assay

Rhamnolipid production was assessed in the presence and absence of drugs using the oil spreading method described previously [25]. Briefly, 20 µL of crude oil was transferred to the surface of 15 ml distilled water in a Petri dish forming a thin oily layer on the water surface. Ten µL of the strain cell-free supernatant, with and without drug (at 1/4 and 1/8 MIC) were added to the center of the oily layer. The diameters of the clear zones that correlate to bio-surfactant activity of rhamnolipids were measured.

### Protease inhibition assay

The proteolytic activity was determined using the skimmed milk agar assay described previously [26]. Briefly, MHA plates with 5% skimmed milk were prepared. Overnight culture of *P. aeruginosa* prepared in the presence and absence of the drugs was centrifuged at 4180 × *g* for15 min, and 100 μl aliquots of the supernatants were delivered into cups placed in the skimmed milk agar plates. The clear zone around the cups was measured after incubation at 37 °C for 24 h.

### Hemolysin inhibition assay

The PAO1 strain was cultured in TSB with and without sub-MIC of the tested drug until they reached the post exponential phase (OD_600_ of 2.5; equivalent to 1 × 10^9^ CFU ml^−1^). The cultures were centrifuged at 3520 × *g* at 4 °C. Aliquots of 100 μl of culture supernatant were brought up to 1 ml in hemolysin buffer solution (0.145 mol l^−1^ NaCl, 0.02 mol l^−1^ CaCl_2_) then 25 μl of defibrinated rabbit blood was added and incubated at 37 °C for 15 min. The hemoglobin-containing supernatant was obtained by centrifugation at 5500 × *g* at room temperature for 1 min and A_543_ measured. The hemolytic activity of the drug-free supernatant was considered as 100% hemolysis, and the % of hemolysis in the presence of the drug was calculated compared to that control [27]. All experiments were performed in triplicate, and the average of the three independent readings were taken as the result.

### Molecular docking

The crystal structures of *P. aeruginosa* LasR (PDB code: 2UV0/1.80 Å) [28], RhlR (PDB code: 8DQ0/ 3.74 Å) [29], PqsR (PDB code: 4JVD/ 2.95 Å) [30], and LasB (PDB code: 3DBK/1.40 Å) [31] were retrieved from the Protein Data Bank (https://www.rcsb.org/). The receptor structures were prepared using the QuickPrep protocol in with Molecular Operating Environment (MOE 2019.012) with Amber10: EHT forcefield [32]. Structures of phenothiazine and miconazole were obtained from the PubChem database (https://pubchem.ncbi.nlm.nih.gov/ accessed on 22 June 2023) as canonical SMILES. Each drug structure was prepared through energy minimization using 0.1 Kcal/mol/Å² gradient RMS, and protonation at physiological pH (7.4). Drugs were docked using Alpha triangle placement with the Amber10: EHT forcefield.

### Assessment of virulence gene expression using quantitative real time-PCR (qRT‑PCR)

The PAO1 strain was grown overnight at 37 °C in LB broth with and without 1/8 MIC of miconazole and phenothiazine until the bacteria reached mid log phase (OD_600_ 0.5–0.6). The culture was centrifuged at 6000 × *g* for 15 min, RNA was extracted from the pellet using the GeneJET RNA Purification Kit (Thermo Fisher scientific Inc., Germany) following the manufacturers’ instructions. Reverse transcription followed by qRT-PCR of the QS-genes *lasI, lasR, rhlR and pqsR* followed the protocol described in the SensiFAST™ SYBR® Hi-ROX One-Step Kit (Bioline, UK). The qRT-PCR analysis in the StepOne RT- PCR thermal cycler (Applied Biosystem, USA) used the primers listed in Table 1 [33]. The housekeeping gene *gyrA* served as a reference gene for normalizing gene expression. The relative gene expression in treated strains was compared to their expression in untreated ones using the 2^−∆∆CT^ method [34].

**Table 1:** Primers used in qRT-PCR

Gene name	Primer sequence (5′ → 3′)
*lasI* (F)	CGCACATCTGGGAACTCA
*lasI* (R)	CGGCACGGATCATCATCT
*lasR* (F)	CTGTGGATGCTCAAGGACTAC
*lasR* (R)	AACTGGTCTTGCCGATGG
*rhlR* (F)	GCCAGCGTCTTGTTCGG
*rhlR* (R)	CGGTCTGCCTGAGCCATC
*pqsR* (F)	CTGATCTGCCGGTAATTGG
*pqsR* (R)	ATCGACGAGGAACTGAAGA
*gyrA* (F)	CGAGAAGCTGCTCTCCGAAT
*gyrA* (R)	TCCTCACGGATCACCTCCAT

### Mice survival assay

The influence of miconazole and phenothiazine on the pathogenesis of *P. aeruginosa* was investigated using the mice survival in vivo model described previously [35]. Ethical standards for the animal study were approved by Zagazig University Institutional Animal Care and Use Committee. Briefly, *P. aeruginosa* PAO1 was grown in LB overnight with and without 1/8 MIC of miconazole and phenothiazine. The bacterial suspension was adjusted to a cell density of about 2.5 ×10^7^ CFU ml^−1^ in phosphate-buffered saline (PBS) which was used to inoculate animals.

The experiment included five groups, each consisting of 5 three-weeks-old healthy female albino mice (*Mus musculus*) with comparable weight. In the test groups, mice were injected intra-peritoneally with 100 µl of drug-treated bacteria in sterile PBS, in the third group animals were injected with 100 µl of untreated bacteria (positive control). Two negative control groups are included; one injected with 100 µl of sterile PBS (the 4^th^ group) and the other un-inoculated (the 5^th^ group). All groups were sustained with normal feeding and aeration at room temperature. Every day for 3 successive days, the survivors in each group were recorded.

### Statistical analysis

A one-WAY ANOVA test (Graph Pad Prism 5) was used to determine the significance of the inhibitory activities of drugs against the various *Pseudomonas aeruginosa* PAO1 virulence factors. *P* values < 0.05 were considered statistically significant. The results for in vivo experiment were calculated using Log-rank test, Graph Pad Prism 5 and plotted using the Kaplan-Meier method.

## Results

### The MIC of miconazole and phenothiazine

Miconazole and phenothiazine inhibited the growth of tested isolates at 0.15 and 10 mg ml^−1^, respectively. The inhibitory effect of miconazole and phenothiazine against virulence factors of PAO1 was tested at a sub-MIC (1/4 and 1/8 MIC), corresponding to approximately 0.038 and 0.019 mg ml^−1^ for miconazole and 2.5 and 1.25 mg ml^−1^ for phenothiazine.

### The effect of sub-MIC of miconazole and phenothiazine on *P. aeruginosa* growth

**Fig. 1:**
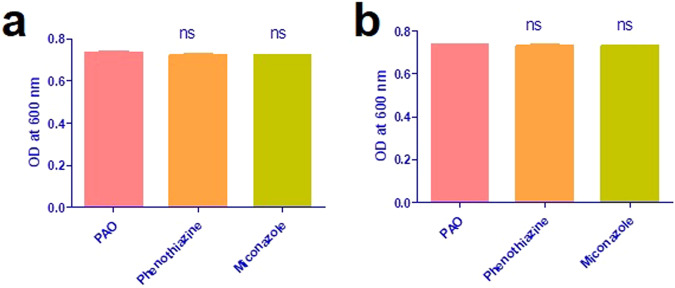
Effect of sub- MIC of tested drugs on *P. aeruginosa* growth. **a** 1/4 MIC (0.0375 mg ml^−1^ of miconazole and 2.5 mg ml^−1^ of phenothiazine) and **b** 1/8 MIC (0.0188 mg ml^−1^ of miconazole and 1.25 mg ml^−1^ of phenothiazine)

To exclude the possibility of sub-MIC miconazole and phenothiazine growth inhibition on *P. aeruginosa*, growth after overnight incubation with/without the drugs was compared by measuring the OD at 600, showing no significant difference between the treated and untreated PAO1 strain. Thus, these sub-inhibitory drug concentrations have no adverse effect on *P. aeruginosa* growth **(**Fig. 1).

### Phenotypic assay of inhibition of *P. aeruginosa* virulence factors

**Fig. 2:**
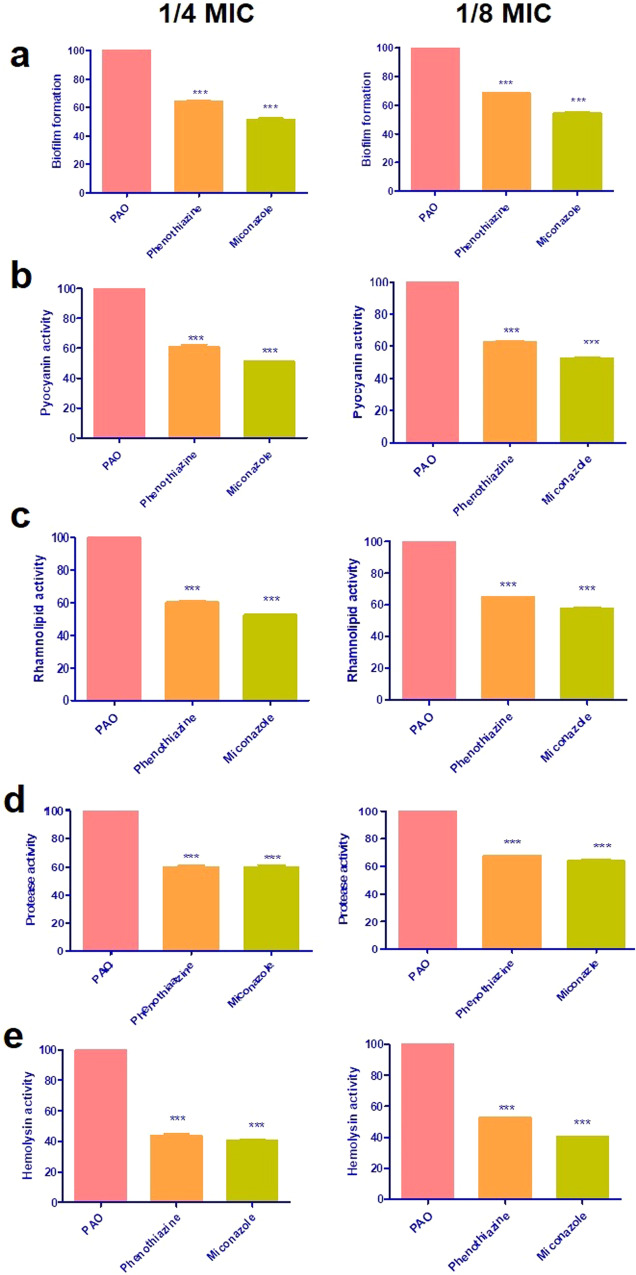
Inhibition of PAO1 virulence factors using sub-MIC of miconazole and phenothiazine. **a** Biofilm formation, **b** pyocyanin production, **c** rhamnolipid activity, **d** protease activity, **e** hemolytic activity. The inhibition was assessed at 1/4 MIC (0.0375 mg ml^−1^ of miconazole and 2.5 mg ml^−1^ of phenothiazine) and 1/8 MIC (0.01875 mg ml^−1^ of miconazole and 1.25 mg ml^−1^ of phenothiazine). The data shown represent the means ± standard errors of the mean (SEM). One-WAY ANOVA test followed by Dunnett’s Multiple Comparison Test

Miconazole and phenothiazine exhibited a significant inhibition of biofilm activity. At 1/4 MIC, miconazole inhibited biofilm formation by 48%, while phenothiazine resulted in approximately 35% inhibition. At 1/8 MIC, miconazole and phenothiazine inhibited biofilm formation by 45% and 31%, respectively (Fig. 2a, Supplementary Fig. 1). Miconazole and phenothiazine reduced the pyocyanin production in PAO1 strain by 49% and 39%, respectively at 1/4 MIC, and 48% and 38%, respectively at 1/8 MIC (Fig. 2b).

Miconazole and phenothiazine treated cultures showed a significant reduction in rhamnolipid activity (Fig. 2c**)**, reduced by 47.5% for miconazole and 40% for phenothiazine at 1/4 MIC, and by 42.5% and 35% at 1/8 MIC, respectively. Inhibition by miconazole ranged between 40% and 36% at 1/4 MIC and 1/8 MIC, respectively, whereas that caused by phenothiazine was 40% at 1/4 MIC and 32% at 1/8 MIC, as compared to the untreated *P. aeruginosa* strain (Fig. 2d).

Miconazole had comparable inhibitory effect at 1/4 and 1/8 MIC, inhibiting hemolysin activity by approximately 59%, whereas phenothiazine decreased hemolysin activity by 56% and 47.5% at 1/4 and 1/8 MIC, respectively (Fig. 2e). Table 2 shows a comparison of inhibitory effect of miconazole and phenothiazine on the phenotypic assay of virulence factors.

**Table 2:** The percentage inhibition of virulence factors using sub-MIC of miconazole and phenothiazine

Assessed virulence factors	Miconazole		Phenothiazine	
	1/4 MIC	1/8 MIC	1/4 MIC	1/8 MIC
Biofilm	48%	45%	35%	31%
Pyocyanin	49%	48%	39%	38%
Rhamnolipids	47.5%	42.5%	40%	35%
Protease	40%	36%	40%	32%
Hemolysin	59%	59%	56%	47.5%

### In silico analysis of interaction of miconazole/phenothiazine with *P. aeruginosa* QS receptors

**Fig. 3:**
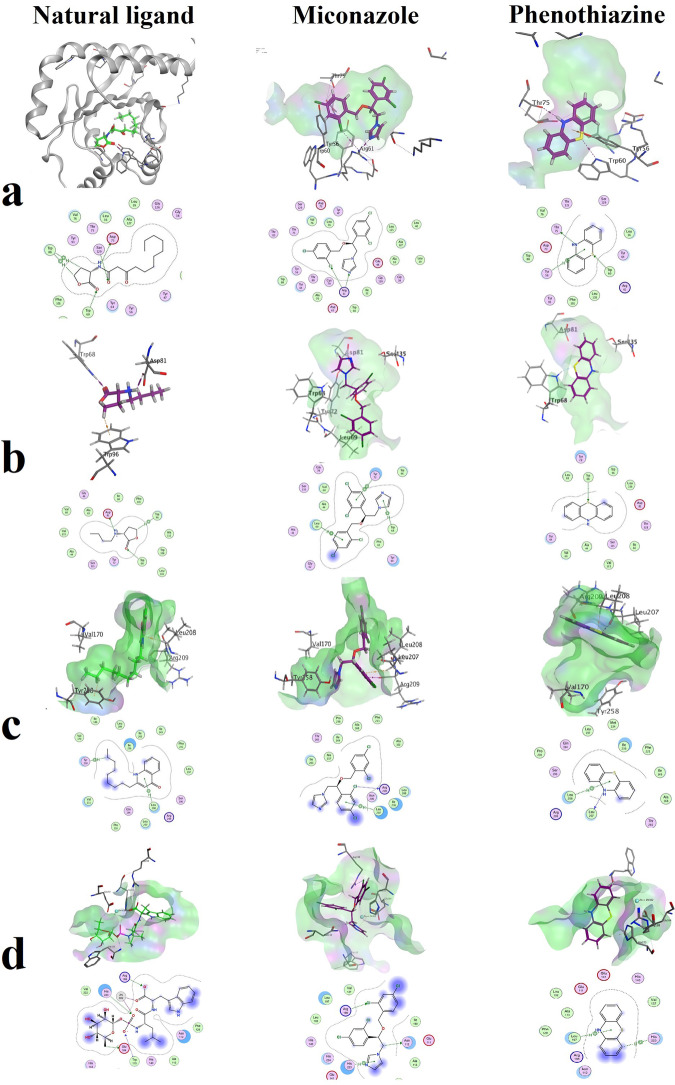
Docking analysis showing the putative binding modes (2D and 3D) of natural ligands, phenothiazine and miconazole into the QS-receptors. **a** LasR natural ligand 3-oxo-C12-HSL had a binding score of −7.778 Kcal/mol, miconazole −9.069 Kcal mol^−1^, phenothiazine −6.129 Kcal mol^−1^. **b** RhlR natural ligand *N*-butyryl-l-homoserine lactone (C4HSL) had binding score −5.796 Kcal mol^−1^, phenothiazine and miconazole showed comparable docking scores of 5.044 and −6.613 Kcal mol^−1^, respectively. **c** PqsR natural ligand NHQ had a docking score −6.456 Kcal mol^−1^, while phenothiazine and miconazole had scores of −5.410 and −6.485 Kcal mol^−1^, respectively. **d** LasB ligand phosphoramidon had a docking score of −12.919 Kcal mol^−1^, with lower scores of −4.346 Kcal mol^−1^ for phenothiazine and −6.018 Kcal mol^−1^ for miconazole. The ligand and active site amino acids are highlighted in blue and cyan, respectively, show strong hydrophobic/ hydrophilic interactions

Molecular docking provided insights into the possible molecular interactions of phenothiazine and miconazole with *P. aeruginosa* QS receptors. *P. aeruginosa* LasR (PDB code: 2UV0), co-crystalized with the natural auto-inducer 3-oxo-C12-HSL had a docking energy score of −7.778Kcal/mol. Miconazole showed a better binding energy score (−9.069 Kcal mol^−1^) than the natural auto-inducer, while phenothiazine had a slightly higher (−6.129 Kcal mol^−1^) energy score. For *P. aeruginosa* RhlR, the natural autoinducer *N*-butyryl-l-homoserine lactone (C4HSL) had comparable docking scores with phenothiazine and miconazole (S = −5.044 and −6.613 Kcal mol^−1^ respectively), but miconazole better filled of the hydrophobic active site. Several potential arene interactions could have resulted in a slightly better score for miconazole, however phenothiazine was able to mimic the auto-inducer in hydrogen bonding with Trp68 (Fig. 3, Supplementary table 1).

For *P. aeruginosa* PqsR (PDB code: 4JVD), the co-crystalized natural ligand 2-nonyl-4-hydroxy-quinoline (NHQ) had docking energy score of −6.456 Kcal mol^−1^. Although, docking scores of both phenothiazine and miconazole were comparable (−5.410 and −6.485 Kcal mol^−1^, respectively), phenothiazine could only fill pocket B of the active site. Miconazole, on the other hand, could orient itself in a conformation to fill both pockets A and B, in close proximity to the key amino acid Leu208 and forming extra hydrogen bonding with Arg209 pulling the imidazole ring further away from Tyr258 but still fitting perfectly in the hydrophobic active site (Fig. 3).

The zinc dependent metallopeptidase LasB (PDB code: 3DBK) with its inhibitor phosphoramidon had a docking energy score of −12.919 Kcal mol^−1^, however, neither phenothiazine or miconazole had any theoretical interaction with the zinc metal, leading to less favorable enzyme-ligand interaction with S scores of −4.346 Kcal mol^−1^ for phenothiazine and −6.017 Kcal mol^−1^ for miconazole (Fig. 3, Supplementary table 1).

### Miconazole and phenothiazine reduced virulence gene expression

**Fig. 4:**
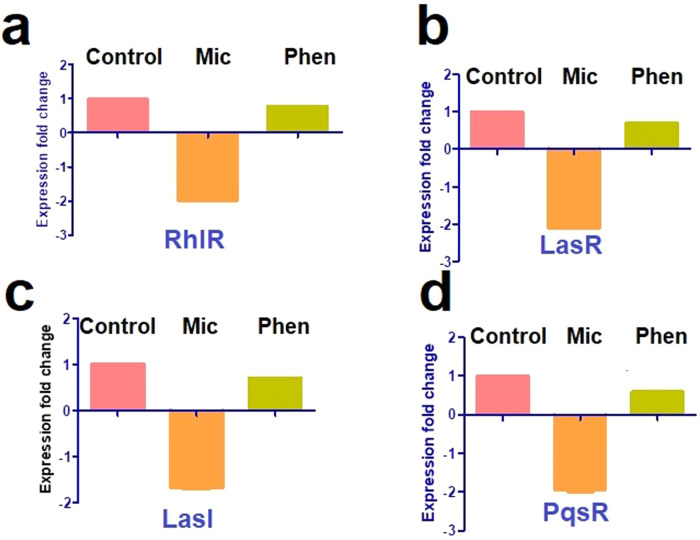
Down-regulation of *P. aeruginosa* QS genes. **a** RhlR, **b** LasR, **c** LasI, **d** PqsR. RNA was extracted from *P. aeruginosa* untreated (control) or treated with sub-MIC of miconazole or phenothiazine, and used for cDNA synthesis. cDNA was amplified by qRT-PCR and changes in the expression of each QS gene normalized in relation to Ct values of the housekeeping gene *gyrA*. Fold change in gene expression in miconazole- and phenothiazine-treated *P. aeruginosa* was calculated using the 2^−ΔΔCT^ method. The data shown are the mean ± SEM from three experiments. *P* < 0.05 was considered significant from the one-way ANOVA test. The tested drugs significantly decreased the expression of lasI, lasR, rhlR and pqsR (*p* < 0.0001)

The influence of miconazole and phenothiazine on the expression of QS-encoding genes was evaluated by qRT-PCR. The expression levels of rhlR, lasR, lasI, and pqsR were reduced after PAO1 treatment with sub-MIC levels of miconazole (1.8- 2.1-fold reduction) compared to the untreated PAO1 strain. There was lower reduction (0.2–0.4-fold reduction) in QS-gene expression in phenothiazine treated cells (Fig. 4).

### Miconazole and phenothiazine reduced the pathogenesis of *P. aeruginosa* in vivo

**Fig. 5:**
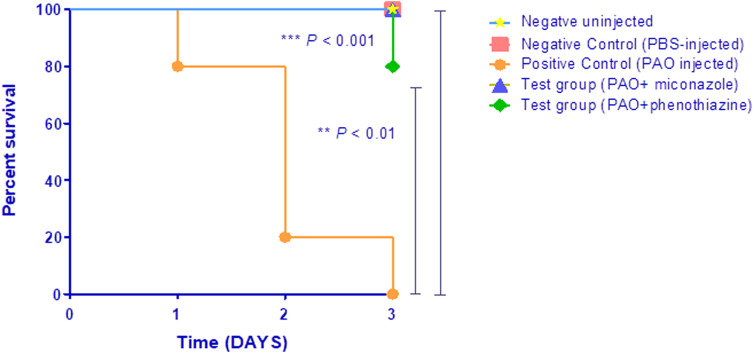
Miconazole and Phenothiazine reduced *P. aeruginosa* pathogenesis in the mouse infection model. Five groups, composed of 5 mice each, were included in the study: two negative control groups either uninfected or injected with sterile PBS, a positive control group was injected with untreated PAO1, and the last 2 groups were injected with PAO1 treated with sub-MIC concentrations of either miconazole or phenothiazine. Mice survival was observed for 72 h, and plotted using the Kaplan–Meier method, and the significance (*p* < 0.05) calculated using a Log-rank test (GraphPad Prism 8). No deaths were observed in negative controls, while no survivors were recorded in the positive control group. Phenothiazine conferred 80% protection, and miconazole showed 100% protection, as all mice survived until the end of experiment (Log rank test for trend *p* = 0.0019). **p < 0.01

The 5 mice injected with untreated PAO1 started to die after 24 h, and all died by the end of the experiment. All the animals (100%) in the control groups (injected with saline or un-injected) remained alive throughout the experiment period. The groups injected with sub-MIC of phenothiazine-treated PAO1 showed improvement in survival rates (80% survival), and complete survival (100%) for the group injected with miconazole-treated PAO1 (Fig. 5).

## Discussion

*P. aeruginosa* is a well-recognized opportunistic pathogen involved in nosocomial infections, especially in patients with a suppressed immune system such as cancer and diabetic patients [36]. This pathogen has an extraordinary capacity to infect different body parts, including invasive infections, which lead to lengthy hospital stays and high mortality [37]. Further, *P. aeruginosa* has developed multiple drug resistance (MDR) and persister strains, which strongly limits the treatment options and represent a major threat worldwide [38, 39]. A recent strategy, used to overcome bacterial resistance, targets virulence factors instead of killing the bacteria. The anti-virulence approach places a lower selective pressure on bacteria, which are then less likely to induce drug resistant phenotypes [1].

In this study, miconazole and phenothiazine were tested for their *P. aeruginosa* ant-virulence potential. Miconazole at 1/4 and 1/8 MIC did not alter the growth and viability of *Staphylococcus aureus* [14], and metformin at sub-MIC has been used to evaluate the anti-virulence activity in a PAO1strain, thus avoiding impact on viability and growth [40]. The MICs of each drug established their sub-lethal levels (1/4, 1/8 MIC) for anti-virulence studies, showing that miconazole was able to inhibit *P. aeruginosa* growth at a lower concentration (0.15 mg ml^−1^) than phenothiazine (10 mg ml^−1^).

Miconazole increases polymyxin-B antibacterial activity against *P. aeruginosa* and other pathogens, reducing polymyxin-MIC from 4-fold to 100-fold [41]. The anti-bacterial activity of miconazole is mainly attributed to the imidazole moiety [42], and in the case of *P. aeruginosa* the inhibition of bacterial flavo-haemoglobins which play an important role in protecting bacterial cells from reactive oxygen species [43]. According to Nehme and coworkers, phenothiazine showed antibacterial activity against Gram negative bacteria, with MIC of more than 1 mg ml^−1^ against *P. aeruginosa* [44]. Phenothiazines are efflux pump inhibitors and their antibacterial activity is attributed to bacterial DNA-intercalation [19].

In the present study, investigating the anti-virulence activity showed that miconazole has the greatest inhibitory effect in comparison with phenothiazine against all the assessed virulence factors as indicated in Table 2. Similarly, D’Angelo and coworkers show that the antifungal drugs clotrimazole and miconazole have anti-virulence potential against *P. aeruginosa* using an in silico model and qRT-PCR [20]. However, D’Angelo study also stated that these drugs did not inhibit PAO1 growth in MHB even at the highest concentration achievable in solution. Another study illustrates that miconazole significantly inhibits the virulence of the Gram-positive pathogen *S. aureus*, including its biofilm formation (86–90%), hemolysin activity (79.5- 82%) and lipase activity (20–25%) [14]. Similarly, the antifungal drug micafungin significantly suppresses the level of QS-controlled virulence factors including pyocyanin, rhamnolipid and biofilm formation in a *P. aeruginosa* standard strain [45].

On the other hand, phenothiazine had a smaller impact against *P. aeruginosa* virulence, but still significant inhibitory activity. With the greatest inhibitory action against hemolysin (47–56%), and the lowest inhibition against biofilm formation (31–35%) as indicated in Table 2. Consistent with our results, phenothiazine and other antipsychotic drugs (thioridazine and chlorpromazine) inhibit biofilm formation, elastase, and pigment production of *P. aeruginosa*, mediated by QS-dependent gene expression [19].

QS systems control the production of an array of virulence factors and biofilm formation in diverse bacterial pathogens, and are thus considered ideal targets for anti-virulence therapy [46]. We thus investigated the ability of miconazole and phenothiazine to impair QS-gene expression using qRT-PCR, with that of *lasI, lasR, rhlR* and *pqsR* genes significantly reduced (1.8–2.1-fold reduction) in the presence of miconazole but less so for phenothiazine (0.2–0.4-fold reduction). Similarly, the antifungal drug clotrimazole reduces the mRNA level of *pqs*-controlled genes [20] and the plant-based inhibitors cinnamaldehyde and salicylic acid significantly downregulate QS-gene expression in the PAO1 strain [47]. Specifically, cinnamaldehyde inhibits *lasI* and *lasR* levels by 13- and 7-fold, respectively, and salicylic acid by 3- fold and 2-fold, respectively [47]. Recently, it was reported that the analgesic drug naproxen down-regulates the *lasI* and *rhlI* genes expression and attenuates *Pseudomonas* protease, hemolysin, pyocyanin, biofilm, and motility [48].

In silico analysis was used to support our hypothesis that miconazole and phenothiazine interact with QS receptors. Molecular docking was performed to investigate the type of interactions between our drugs and QS-proteins and to calculate the docking scores which approximately indicate the binding affinity of the drug to receptor protein. A greater negative score denotes a higher likelihood of a more stable binding [49]. It is important to mention that absolute result of molecular docking is not indicative. Only relative binding and comparison with known ligands using consistent parameters and calculations provides perception towards the binding mode of the ligand of interest [50].

Molecular docking with the LasR protein showed that miconazole had stronger theoretical (−9.069 Kcal mol^−1^) in comparison to its natural ligand (−7.778), while phenothiazine was weaker −6.129. For LasB, both phenothiazine (−4.346 Kcal mol^−1^) and miconazole (−6.018 Kcal mol^−1^) had weaker theoretical binding than its natural ligand (−12.919 Kcal mol^−1^). For the RhlR receptor, phenothiazine, miconazole and its natural ligand (C4HSL) had docking scores of −5.044, −6.613 and −5.796 Kcal mol^−1^, respectively. Finally, with PqsR, the natural ligand NHQ, miconazole and phenothiazine had comparable docking scores of −6.456, −6.485 and −5.410 Kcal mol^−1^, respectively. Collectively, the in silico analysis supports the qRT-PCR results showing that miconazole is a more potent inhibitor of QS-genes than phenothiazine, with superior activity in LasR Binding.

D’Angelo and coworkers reported similar molecular docking results [20], with antifungal drugs clotrimazole and miconazole able to bind to the PqsR regulator, with putative binding values of −8.4 and −8.5 Kcal mol^−1^, respectively, slightly lower than that of the natural ligand NHQ ( − 7.9 kcal/mol). From the qRT-PCR and molecular docking results, it is reasonable to propose that miconazole anti-virulence action is mediated QS-regulator inhibition. As we mentioned before that Las signal control the QS-circuit that activates both Rhl and Pqs [4], this mean the strong binding capacity of miconazole to LasR could contribute to the down-regulation of the other QS-signals. While phenothiazine had a seemingly lower theoretical impact on QS-proteins, it may also affect other cellular targets such as efflux-pumps as previously reported [51, 52].

To underscore the clinical relevance of the drugs, the anti-virulence potential of miconazole and phenothiazine was evaluated in vivo. Mice injected with miconazole-treated PAO1 showed 100% survival, similar to the negative control group, while those injected with phenothiazine-treated PAO1 had 80% survival. Similarly, the FDA-approved drugs clofoctol and raloxifene protected *Galleria mellonella* larvae from *P. aeruginosa* strains, attenuating its lethality [20, 53] just as cinnamaldehyde in combination with gentamicin rescued the nematode *Caenorhabditis elegans* from PAO1 infection and improved its survival rate by 54% [54]. In addition, the QS-inhibitor drugs erythromycin, azithromycin and propranolol induced 80–100% survival in mice injected with *Acinetobacter baumannii* [16]

Collectively, phenotypic analysis indicated that miconazole and phenothiazine have inhibitory activity against *P. aeruginosa* virulence factors, including biofilm formation, pyocyanin, protease, rhamnolipid and hemolysin production, with miconazole having a greater impact. Genotypic analysis and molecular docking predict miconazole to be a better inhibitor of QS-receptors than phenothiazine. The in vivo study demonstrates 100% survival in mice injected with miconazole-treated *P. aeruginosa*.

One of the raised concerns about FDA-approved drug repurposing is the concentrations of these drugs do not always meet the pharmacological limits for human use. To compensate this shortage in reaching the therapeutic plasma level with these drugs, it could be possible to use these drugs in topical formulations for skin and soft-tissue infections or as aerosols in respiratory infection. In addition, these drugs could be used at lower concentration in combination with antibiotics. Taken together, these data indicate that miconazole is a promising anti-virulence agent with strong clinical potential to treat infections caused by resistant *P. aeruginosa* strains in combination with antimicrobial drugs.

### Supplementary information

Supplemetary table 1 and figure 1

## Data Availability

The datasets used /or analyzed in the current study are available from the corresponding author on reasonable request.
