# Supplementary material for: Miconazole and phenothiazine hinder the quorum sensing regulated virulence in Pseudomonas aeruginosa
Source: J Antibiot (Tokyo). 2024 May 9;77(7):454–65. doi: 10.1038/s41429-024-00731-5 (PMC11208154; doi:10.1038/s41429-024-00731-5)
Supplement: Supplementary file 1 — Supplemetary table 1 and figure 1 [file 41429_2024_731_MOESM1_ESM.docx]

**Miconazole and phenothiazine hinder the quorum sensing regulated virulence in** ***Pseudomonas aeruginosa* Supplementary Data**

**
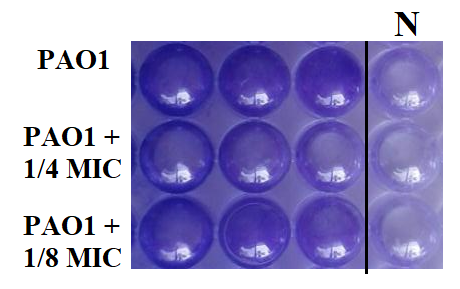
**

**Supplementary figure 1: biofilm inhibition assay (N: negative control)**

**Supplementary Table 1: The binding score and mode of each ligand with the different residues inside the active site of the QS-receptors of *Pseudomonas aeruginosa***

| **QS receptor** | **Ligand** | **Binding Score** | **Interactions** |
| --- | --- | --- | --- |
| LasR | 3-oxo-C12-HSL | -7.7783 | H bond: Trp60, Asp73, Ser129  Pi interactions: Trp88 |
|  | Phenothiazine | -6.1294 | H bond: Trp60, Thr75  Pi interactions: Trp88, Phe101, Ser129 |
|  | Miconazole | -9.0694 | Tyr56 |
| RhlR | C_4_HSL | -5.7957 | Asp84, Trp68, Trp96 (Pi) |
|  | Miconazole | -6.6126 | Tyr64, Leu69, Tyr72 |
|  | Phenothiazine | -5.0438 | Trp68 |
| PqsR | NHQ | -6.4557 | Leu208, Tyr258 |
|  | Miconazole | -6.4850 | Leu207, Arg209 (H bond) |
|  | Phenothiazine | -5.4102 | Leu208, Leu207 (H bond) |
| LasB | Phosphoramidon | -12.9192 | Ionic interaction: Zinc metal.  H bond: Trp 115, Glu 164, Arg 198,nHis 223 |
|  | Miconazole | -6.0179 | Hydrogen bond: Asn 112, Arg 198.  H-pi interactions: His 223 |
|  | Phenothiazine | -4.3462 | Leu 197, His 223 |
